# Proteotoxicity and Neurodegenerative Diseases

**DOI:** 10.3390/ijms21165646

**Published:** 2020-08-06

**Authors:** Clara Ruz, Jose Luis Alcantud, Francisco Vives Montero, Raquel Duran, Sara Bandres-Ciga

**Affiliations:** 1 Instituto de Neurociencias Federico Olóriz, Centro de Investigación Biomédica, 18016 Granada, Spain; clararuz@ugr.es (C.R.); jlalcantud@ugr.es (J.L.A.); fvives@ugr.es (F.V.M.); rduran@ugr.es (R.D.); 2 Molecular Genetics Section, Laboratory of Neurogenetics, National Institute on Aging, National Institutes of Health, Bethesda, MD 20892-3707, USA

**Keywords:** proteotoxicity, Alzheimer’s disease, Parkinson’s disease, Amyotrophic Lateral Sclerosis, Huntington disease

## Abstract

Neurodegenerative diseases are a major burden for our society, affecting millions of people worldwide. A main goal of past and current research is to enhance our understanding of the mechanisms underlying proteotoxicity, a common theme among these incurable and debilitating conditions. Cell proteome alteration is considered to be one of the main driving forces that triggers neurodegeneration, and unraveling the biological complexity behind the affected molecular pathways constitutes a daunting challenge. This review summarizes the current state on key processes that lead to cellular proteotoxicity in Alzheimer’s disease, Parkinson’s disease, Huntington’s disease, and amyotrophic lateral sclerosis, providing a comprehensive landscape of recent literature. A foundational understanding of how proteotoxicity affects disease etiology and progression may provide essential insight towards potential targets amenable of therapeutic intervention.

## 1. Introduction

Proteotoxicity, meaning impairment of cellular function as a result of protein misfolding or aggregation, is a major hallmark of a broad range of neurodegenerative diseases, including Alzheimer’s disease (AD), Parkinson’s disease (PD), amyotrophic lateral sclerosis (ALS), frontotemporal dementia (FTD), and Huntington’s disease (HD), among others. A common denominator in the etiology of these diseases is that neuronal damage is frequently caused by abnormal aggregation and deposition of proteins in which altered specific molecular mechanisms lead to cell toxicity and degeneration.

Aging constitutes the main risk factor for neurodegenerative diseases [1] and proteotoxicity is an enhanced occurrence during the aging process. As we age, the ability of our organism to maintain protein homeostasis substantially decreases. Several studies have proven that the aging process actively suppresses our capacity to clear toxic protein aggregates, promoting the accumulation of these aggregates and initiating neurodegeneration late in life [2]. In general, aggregates of toxic proteins originate when soluble proteins gradually become insoluble filamentary polymers. These filamentous structures accumulate in the form of fibrils and end up depositing and aggregating in the nucleus, cytoplasm, or extracellular space of affected brain cells [3]. Eukaryotic cells have essential protein quality control (PQC) systems, including chaperones that safeguard proteins against aggregation, the ubiquitin-proteasome system (UPS) and the autophagy-lysosome system that act in the degradation of misfolded proteins. A decrease activity or saturation of these proteolytic systems results in neuronal impairment and cell death.

The fact that proteotoxicity is a shared emergent pattern among these diseases raises the question of whether common therapeutic interventions aimed at targeting dysregulated proteins by either blocking over aggregation or accelerating degradation may be applicable. Although it is widely known that similar molecular pathways altering cell viability through protein deposition cause these disorders (Table 1), the reality is that elimination of proteins in a way that toxicity can be mitigated is more complex than expected.

In this review, we explore underlying mechanisms that lead to proteotoxicity in four main neurodegenerative diseases in which this molecular signature plays a major role including HD, ALS, PD, and AD. In the current scenario, we summarize insights on the relationship between proteotoxicity and disease pathogenesis gained along the way, focusing on the distinct mechanisms underlying proteotoxicity in these diseases that have enhanced our needed understanding for the development of future and indispensable disease-modifying therapies. Uncovering how these biological processes trigger and promote disease progression certainly constitutes one of the main challenges in deciphering the pathophysiology of these debilitating conditions.

## 2. Proteotoxicity in Huntington Disease

Huntington disease (HD) is a devastating neurodegenerative disorder caused by the expansion of a CAG trinucleotide repeat within the first exon of the *HTT* gene, which encodes huntingtin. The CAG repeat generates an expanded polyglutamine (polyQ) tract in the terminal region of the protein that misfolds to adopt a pathogenic conformation [87].

### 2.1. Structure of the Huntingtin Protein

Huntingtin is a large well-conserved protein of 3144 amino acids whose structure consists of many helical repeat units. The N-terminal region has been extensively studied, as it contains the expandable polyQ tract. The polyQ tract is followed by a proline-rich domain that is thought to be critical for stabilizing the structure of the polyQ stretch as well as taking part in protein–protein interaction mechanisms [88]. The rest of the protein includes other functional motifs such as the elongation factor 3, protein phosphatase 2A, and TOR1, which although they have been less well-characterized may act regulating the huntingtin function or localization [89]. In the population not affected by HD, the CAG sequence is repeated between 9 and 35 times, with an average median that ranges between 17 and 20 repeats [90]. When the CAG expansion exceeds 35 repeats proteotoxicity arises originating HD. Proteotoxicity is inversely proportional to the length of the CAG expansion, with juvenile onset being associated with about 75 or more repeats.

### 2.2. Physiological Role of the Huntingtin Protein

The huntingtin protein has long been considered having no clearly defined cellular function. Recent studies aimed at elucidating its role on disease have revealed that the protein acts controlling the anterograde and retrograde transport of organelles. Huntingtin mediates endocytosis, vesicle recycling, and endosomal trafficking. It acts regulating transport efficacy and directionality, including trafficking of precursor vesicles [91], endosomes and lysosomes [92,93], autophagosomes [94], and neurotrophic and neurotransmitter factors-containing vesicles [95,96]. The protein is also thought to coordinate cell division [97] and influence organogenesis and tissue maintenance by regulating metabolism. It is essential for embryonic development and the formation of the nervous system [98].

### 2.3. Pathological Mechanisms of Huntingtin Aggregation

An increase in the size of the CAG segment leads to the production of an abnormally long version of the huntingtin protein. The elongated protein is cut into smaller, toxic fragments that bind together and accumulate in neurons, disrupting their biological functions. It is still highly controversial whether the proteotoxicity process underlying HD is due to a gain of toxic function or a loss of function mechanism, or both. The fact that HD is an autosomal dominant disorder argues in favor of a gain of function of the protein. Mutant huntingtin is cleaved by proteases generating N-terminal fragments containing the abnormal polyQ tract, and it has been proven that longer polyQ stretches can promote their self-cleavage [99]. The shorter the fragments are the more toxicity is inferred in the cell where they may cause neuronal cell death by interfering with the transcription process in the nucleus [100]. Although a loss of function effect is also plausible, HD is unlikely to be caused by a straightforward loss-of-function mechanism. It was long thought that inactivation of one of the two wild-type huntingtin alleles was not a cause for HD phenotype [101]. However, animal models suggest that a loss-of-function of the wild-type huntingtin protein may contribute significantly to several components of disease pathology. Homozygous and heterozygous knockout models in mice and drosophila have shown neurodevelopmental defects and behavioral changes [102,103]. A loss of normal huntingtin function may have wider ranging impacts on cell physiology than previously thought, but this may be more observed in the aging process. Lower levels of wild-type huntingtin have been linked to proteasome and autophagy inhibition as well as vesicle transport impairment and signaling altered mechanisms related to the production and transport of the brain-derived neurotrophic factor (BDNF) [100].

### 2.4. Therapeutic Insights

Although there is currently no cure for HD, recent advances in genetic therapies hold great promise. Lowering the levels of the toxic mutant huntingtin by targeting either DNA or RNA and preventing protein expression while leaving the wild type copy untouched is currently the most prominent area of research in HD therapies. Recent research conducted in mice, patient-derived fibroblasts, and neurons revealed encouraging results using an engineered, synthetic transcription factor that achieved robust allele-selective targeting of endogenous mutant huntingtin. This approach provided a new method for the field to investigate the role of huntingtin in vivo [104]. Since huntingtin is involved in a wide range of biological functions, and a full knockdown of the gene is lethal in mice, maintaining some expression of the *HTT* gene is critical. Targeting gene regulation has gained a lot of attention in the last years since it is designed to enable precise and long-term repression of the selected gene following a single administration of an adeno-associate virus (AAV) that does not cut or modify the target DNA. This intervention is currently considered as one of the top potential therapeutic avenues since it prevents transcription of toxic huntingtin targeting all the potentially toxic downstream forms of the mutant protein. Another state of the art approach that has garnered a lot of attention recently is using antisense oligonucleotides (ASOs). A recent study showed promising results using ASO mediated suppression of the huntingtin (HTT) protein in the brains of adults with HD [105]. Using this approach, the first human safety trial was completed in 2017 and consisted of a drug developed to reduce the levels of the huntingtin protein in the nervous system, which successfully lowered levels of the toxic protein in participants. This therapy is currently under a major global phase 3 clinical trial in order to test the long-term safety and clinical efficacy as well as whether it slows disease progression or not. The final goal of these therapies is to delay or prevent neurodegeneration while function is still intact, giving gene carriers quality of life without impairment.

## 3. Proteotoxicity in Amyotrophic Lateral Sclerosis

Amyotrophic Lateral Sclerosis (ALS) is a fatal, neurodegenerative disease characterized by the death of both the upper and lower neurons of the spinal cord, responsible for controlling voluntary muscles [106].

A considerable number of proteins play a major role in ALS etiology, including Superoxide Dismutase 1 (SOD1), Fused in Sarcoma (FUS), Ataxin-2, and TANK-binding kinase 1 (TBK-1). In the current review we focus on the most relevant pathological hallmark: TAR DNA-binding protein 43 (TPD-43) [107]. TDP-43 is a highly conserved protein which belongs to the nuclear ribonucleoprotein family (hnRNP). It binds RNA and regulates mRNAs involved in the development of neurons [108].

### 3.1. Structure of the TDP-43 Protein

TDP-43 is a 414 amino acid protein predominantly localized in the nucleus but also in the cytoplasm and mitochondria. In ALS there is an increase in the cytoplasmic concentration via aggregation. The N-Terminal Domain (NTD) of the protein is responsible of TDP-43 dimerization. Research has pointed that this domain plays a major role on its physiological functions, being responsible for its aggregation [109]. TDP-43 also possesses two RNA Recognition Motifs (RRMs) with high specificity towards UG/TG-rich sequences of RNA/DNA molecules, given its role in several RNA processes. Since it has multiple locations, TDP-43 has different localization signals, such as: Nuclear Export Signal (NES), Nuclear Localization Signal (NLS), and Mitochondrial Localization Motifs (M1, M3, and M5). The C-Terminal Domain (CTD) is crucial for TDP-43 pathological behavior. This domain is aggregation-prone and contains phosphorylation targets as well as enzymatic caspase activity, which can produce highly cytotoxic C-terminal fragments [107].

### 3.2. Physiological Functions of TDP-43

TDP-43 takes part in plenty of molecular processes, such as RNA metabolism by regulating splicing, RNA maturation, stability and trafficking, transcriptional regulation by repressing transcription of some genes like *SP-10*, translational regulation by associating with 3′UTR mRNA targets, protein stability and location, and response to stress. Regarding the former, TDP-43 can be recruited into stress granules so it stops specific mRNAs from being translated during the cellular stress response [110].

### 3.3. Pathological Mechanisms of TDP-43 Aggregation

TDP-43 has been identified in 97% of ALS patients as aggregated protein in both the form of phosphorylated and ubiquitinated cytoplasmic inclusions. Albeit to a lesser extent, aggregates of acetylated and sumoylated TDP-43 have been also identified. Among all factors that influence its aggregation, post-translational modifications, C-terminal fragments and its prion-like behavior should be highlighted [111].

Post-translational modifications of TDP-43 change protein structure, localization, function, and therefore propensity to aggregate. Ubiquitination of TDP-43 has been shown to increase its nuclear depletion as a result of an incorrect degradation. Hence, its nuclear functions are affected by increased cytoplasmic accumulation which is highly toxic for the cell. Phosphorylation can also enhance its propensity to aggregate and being split into C-terminal fragments (CTF), which in turn leads to increased proteotoxicity [112]. These CTFs have a length between 25—35 kDa, some of them being more toxic than intact TDP-43. These motifs can also co-aggregate with other CTFs and TDP-43, thus increasing its toxic potential. The fact that different CTFs can have variable levels of toxicity and aggregation makes future studies necessary in order to elucidate the relationship between its structure and the cellular damage.

Symptoms of ALS are thought as a combination of several factors triggered by proteotoxicity. Loss of TDP-43 native functions causes failure in energy metabolism leading to protein metabolism, mitochondrial dysfunction, aggravated oxidative stress, alterations in several autophagy lysosomal processes, dysregulation of metal ion homeostasis (mainly zinc, copper, and manganese), impaired axonal outgrowth, or alteration in chromatin dynamics [107]. Additionally, it is thought that these events can be due to a toxic gain of function of aggregated TDP-43, since it is not an inert product. For example, aggregated cytoplasmic TDP-43 can sequester nuclear TDP-43 and other mRNAs proteins involved in transcriptional regulation [113].

### 3.4. Prion-like Behavior of TDP-43 Aggregates

Prions are misfolded proteins with toxic activity which can transmit their misfolded shape onto normal variants of the same protein. Prions are responsible of multiple degenerative diseases, such as Creutzfeldt-Jakob disease or kuru [107]. There are several studies suggesting that ALS might be a ‘prion-like’ disease because of the ability of the C-terminal region of TDP-43 to form stable β-sheets resembling amyloid-like fibrils. It seems that this structure is able to start a seeding mechanism similar to prions [113]. Despite this hypothesis, the mechanisms by which TDP-43 spreads into nearby cells remain unclear. Studies suggest that TDP-43 is transported through the axons, affecting nearby oligodendrocytes and neurons. This theory supports the idea of ALS spreading to neurons, just like prion diseases [112].

### 3.5. Other Proteins Involved in Amyotrophic Lateral Sclerosis

Besides TDP-43, it should be pointed out that FUS is a key proteotoxicity driver. Like TDP-43, FUS is an RNA binding protein (RBP) that regulates RNA metabolism, splicing, transcription, and transportation, and contributes to repair DNA damage. Its pathogenic properties are related to a mislocalization in the cytoplasm and a loss of function mechanism [114]. The structural domains exerting a major effect on ALS pathology seem to be located at the N-terminal prion-like domain, a portion of the glycine-rich region and the C-terminal nuclear localization signal (NLS), which are crucial for FUS mislocalization [27]. Just like TDP-43, such mislocalization leads to an aggregate that is able to sequester proteins and RNA gaining a toxic ability. Nevertheless, there is much controversy about the mechanisms driving this aggregation with some research suggesting that FUS loss of nuclear function is enough to develop toxicity [115]. Approximately 35% of FUS-ALS patients develop the disease before the age of 40 years, and this aggressiveness is thought to be due to the synergy existing between FUS and other RBPs. In 2019, Marrone et al. demonstrated that FUS-ALS is linked to defects in the PQC system, and also that an inhibition of autophagy increases cytoplasmic FUS levels, leading to proteotoxicity. Inducing autophagy restored protein homeostasis showed improvement in FUS levels and cell survival. These autophagy inducer molecules could open a promising scenario in ALS disease-modifying therapies [28].

Another major contributor to proteotoxicity in ALS is SOD1. This enzyme is responsible of catalyzing the inactivation of superoxide into oxygen and hydrogen peroxide, being an indispensable antioxidant defense mechanism present in most of the cells. SOD1 is associated with an autosomal dominant form of ALS. Mutations in *SOD1* destabilize its structure, which may lead to misfolding, oligomerization and formation of aggregates. Due to its ability to decrease chaperone and proteasome activity, these toxic aggregates are responsible for an insufficient clearance of intracellular proteins, which can bring aberrant protein–protein interactions. A lack of native structure cause aberrant redox chemistry which is toxic for the cells, leading to an increased amount of reactive oxygen species (ROS), capable of damaging other macromolecules [116]. Additional toxic features caused by SOD1 pathology include glutamate excitotoxicity due to a reduction in the astroglial glutamate transporter EAAT2 which leads to an excessive calcium influx, thus triggering destructive processes responsible for ALS pathology.

Furthermore, misfolded SOD1 can be localized to the outer membrane and matrix of the mitochondria, leading to malfunction and disruption of ATP production and energy homeostasis. Additionally, mutant SOD1 has been seen to be involved in loss of neurotrophic signaling and defective axonal transport in motor neurons [117]. Misfolded SOD1 can be secreted into the extracellular space and then sequestered by nearby cells where misfolded SOD1 is able to recruit additional misfolded proteins to enlarge itself [118].

Ataxin-2 is a member of the Lsm (Like-Sm) protein family. It is involved in a large number of functions including cellular stress responses and mRNA stability and translation. Ataxin-2 has a polyglutamine tract comprising 22–23 glutamines (Q) in the healthy population. An expansion of 27–33 Qs has been demonstrated to be linked to ALS [119]. Ataxin-2 links TDP-43 and its upregulation increases TDP-43 toxicity. Ataxin-2 is normally located in the cytoplasm of spinal cord neurons, but in ALS patients, its localization is altered, enhancing accumulation that eventually influences TDP-43 mislocalization under stress situations [40].

Finally, TANK-binding kinase 1 is composed of 4 domains: a kinase domain (KD), a ubiquitin-like domain (ULD) and two coiled-coil domains (CCD1 and CCD2). CCD1 is responsible for homodimerization of TBK-1, while CCD2 can bind and phosphorylate optineurin and p62, proteins known to be implicated in autophagy [120]. Several mutations in *TBK1* cause protein truncation and loss of function, leading to a decrease in the protein ability to phosphorylate and bind optineurin and p62. Furthermore, other mutations can drop mRNA and protein levels, resulting in a lower activation of autophagy adaptors, leading to accumulation of toxic protein aggregates in motor neurons [41].

### 3.6. Therapeutic Insights

ALS is a complex and devastating disease with no diagnostic biomarkers or effective treatments to slow down its progression. Current strategies for intervention include preventing TDP-43 from aggregating or improving its clearance through a PQC system via a TDP-43 aggregation inhibitor molecule, such as acridine-imidazole derivative (AIM4) [121], 4-aminoquinoline derivatives [122], or a treatment with several copper complexes which has shown to inhibit TDP-43 accumulation and C-terminal fragment aggregation.

The use of a mutant chaperone Hsp104 has been shown to decrease TDP-43 toxicity, aggregation, and promote nuclear localization [123]. Furthermore, nuclear import receptors as karyopherin-β2 have been proven to prevent aggregation of TDP-43 and other RNA-binding proteins, restoring their localization and functions [124].

Additionally, other ways of treating this disease include molecules targeting mechanisms leading to cellular dysfunction. Some examples are mexiletine that reduces neuronal hyperexcitability [125], edaravone, a free-radical scavenger which decreases oxidative stress [46], or rapamycin, an autophagy inducer that forms autophagosomes enhancing protein degradation, currently in a phase II clinical trial [126].

In the last years, several clinical trials have been carried out in order to test potential novel drugs for this terrible disease. Some examples are ropinirole hydrochloride, a molecule which targets abnormal TDP-43/FUS aggregation. It is a phase I/IIa randomized, double-blind, placebo-controlled, single-centre, open-label continuation trial with expected results by March 2021 [127]; tamoxifen, that showed ability to enhance autophagy and decrease TDP-43 aggregation in a small randomized double-blind trial whose results revealed a slight effect on attenuating disease progression for six months [128]; or arimoclomol, a heat shock protein co-inducer with neuroprotective properties in *SOD1* related ALS, which was tested in a randomized, double-blind, placebo-controlled trial and lacked an important therapeutic benefit [129].

## 4. Proteotoxicity in Parkinson’s Disease

Major advancements in genetics and neuropathology led to the identification of α-synuclein as the major pathological hallmark of PD [130,131]. The *SNCA* gene encodes for a presynaptic protein called α-synuclein, identified as the main component of amyloidogenic protein deposits known as Lewy bodies (LB) and Lewy neurites (LN) in PD [132]. Deleterious genetic variation in *SNCA* results in the accumulation and aggregation of misfolded α-synuclein [133]. Although pathological mechanisms of PD are numerous and diverse, α-synuclein proteotoxicity has been hypothesized to be one of the main contributors to the neurodegenerative process, spreading the pathological condition to neighboring cells like a prion-like disease [134]. In turn, these α-synuclein aggregates can interact to promote disruption of protein homeostasis and thus interfere cell normal topography and physiology including stress formation on the endoplasmic reticulum (ER), mitochondrial dysfunction and defective neurotransmission, which if prolonged, could lead to neuronal death [58,135].

### 4.1. Structure and Physiological Function of α-Synuclein

α-synuclein is a small soluble protein (140 amino acid) highly expressed in the presynaptic zone of neuronal cells [136]. Unlike its pathological behavior, the normal function of this protein remains elusive. Some studies link it with synaptic transmission and vesicle trafficking, mitochondrial homeostasis, gene expression, protein phosphorylation, or even fatty acid binding [137,138]. α-synuclein is differentiated into three main domains: the N-terminal domain (amino acids 1–60); an hydrophobic central domain known as non-amyloid β-component domain or NAC (amino acids 61–95), which is associated to α-synuclein aggregation in humans when β-sheet structure is acquired [139]; and the C-terminal region (amino acids 96–140) suggested to be involved in multiple functions, including self-protection of α-synuclein from aggregation [140,141]. Except for some cases [142], native α-synuclein is commonly found unfolded in a monomeric state, [143,144]. In contrast, there is no controversy under pathological conditions where α-synuclein adopts a β-sheet amyloid conformation, promoting proteotoxic deposits.

### 4.2. Pathologic Mechanisms of α-Synuclein Aggregation

The aggregation process of α-synuclein in vitro follows a similar pattern to other amyloid diseases. The oligomerization follows a nucleation-dependent pathway divided in three phases: the lag phase where the monomers form the aggregation nuclei; the elongation phase in which fibrils tend to grow exponentially, and finally the stationary phase where monomers leakage result in a decrease of growing rate [145,146,147]. Oligomers grow faster into the elongation phase, converted into protofibrils due to the monomer addition which conforms the amyloidogenic mature form. However, although the protein has mechanisms to protect itself from aggregation [140,141], the reason why that occurs has not been identified yet. Thus, potential risk factors have been proposed to initiate/potentiate this procedure, such as genetic mutations [148,149], environmental factors as iron exposure and pesticides and dietary aspects [150].

α-synuclein toxicity follows the prion-like hypothesis where the misfolded protein spreads cell to cell via endocytosis transmission. Subsequent to α-synuclein aggregation, anomalous oligomers are released from cells. Exogenous α-synuclein fibrils can invade inside cells, seeding them into recipient cells, and induce its propagation cell to cell by originating a new cycle of endocytosis. This event would finally end inducing new LB-like inclusions [67]. The presence of α-synuclein deposits, according to similar mechanisms in other neurodegenerative diseases, suggests that the main link between α-synuclein and PD pathology is the gain of function hypothesis. In this direction, we briefly recap the main proteotoxicity effects of α-synuclein in PD pathogenesis.

Post-translational modification including phosphorylation, ubiquitination, and glycosylation, plays an essential role in protein structure, modification, and function. Disruption of these processes has been linked to an intricate cellular proteostasis state within the cell. Accordingly, altered hyper phosphorylation was found in α-synuclein site Ser129 in LBs. Therefore, phosphorylation of Ser129 has evidenced to facilitate cytotoxic transport to the nucleus, being capable of interacting with histones inducing neurotoxicity [45].

In addition, α-synuclein toxicity within the nucleus may implicate the inhibition of histone acetylation, promoting neuronal disruption and cell death [65,151]. Furthermore, amyloidogenic α-synuclein can modify lipid permeabilization, thus altering the structural components of the cell. One potential hypothesis suggests that these oligomers penetrate the cell membranes and reorganize lipid environment, stimulating the flux of surrounding molecules [152]. An alternative theory supports that oligomers could be fused with the membranes acting as an amyloid pore or channel [153,154]. These events have been suggested to enhance extracellular calcium flux within cells so that could be responsible for the cell death of dopaminergic neurons in PD. In a similar way, α-synuclein aggregates disrupt the main protein clearance systems within the cell and therefore mitochondrial function [155]. The autophagy lysosomal pathway (ALP) and the UPS have been associated with PD pathology. In the last decade, genetic perturbation in several genes including *LRRK2, SNCA, LAMP3, GBA*, and *ATP13A2* affecting lysosomal activity have been elucidated [156,157]. In vitro models showed that pathologic α-synuclein blocks macroautophagy by binding key proteins, such as the lysosomal LAMP2 receptor in ALP or the 20S component in UPS [54,55]. Although further studies are needed, these events suggest that α-synuclein drives proteotoxicity by disrupting protein clearance of substrates. Consequently, accumulation of several substrates in addition to toxic oligomers within the cytosol, rise mitochondrial dysregulation associated with complex I activity, which is impaired in the brain tissue of PD patients and in vitro [158].

Despite *SNCA* being the major genetic contributor to PD etiology, genetic mutations in *PINK1* or *DJ1* in mendelian forms of PD result in a mitochondrial perturbation due to a toxic gain of function in the mutated proteins [59]. PINK1 is a kinase protein involved in several events within the mitochondria, including oxidative phosphorylation or mitophagy. According to the proposed mechanisms above mentioned, α-synuclein toxic effects may lead to disrupted mitochondrial dynamics altering electronic potential and facilitating mitochondrial fragmentation, dysfunctional mitophagy or increased mitochondrial ROS levels. Multiple other cellular pathways are affected by α-synuclein, as ER stress promotion which in turn promotes ER-Golgi transport dysfunction and Golgi fragmentation [60,61]. Taking this together, α-synuclein toxicity drives the main pathologic events in PD. However, how α-synuclein triggers those pathological events in PD, being either cause or consequence of it, remains unclear.

### 4.3. Therapeutic Insights

Since protein aggregation is responsible for neurodegeneration, there is now emerging research towards restoring proteostasis through stimulation of the PQC system. Within the wide range of experimental studies, one of the most promising treatments is ambroxol, a small molecule chaperone that crosses the blood–brain barrier and helps defective GBA protein refold and traffic correctly to lysosomes. In a recent open-label clinical trial of 17 PD patients both with and without *GBA* mutations, ambroxol increased GBA activity and modulated α-synuclein levels in cerebrospinal fluid [159]. Although a larger study is required, ambroxol therapy seems to bring hopeful results. Regarding immunotherapy, there are several studies in preliminary stages of clinical investigation [160]. The monoclonal antibody PRX002, designed to preferentially target soluble and insoluble aggregated forms of a-synuclein, significantly reduced free serum α-synuclein levels in humans, [161] being safe and well tolerated by participants after intravenous infusions of multiple ascending doses [162]. BIIB054, another anti a-synuclein antibody, has demonstrated an acceptable safety among participants, and has been currently enrolled in novel trials to examine its capacity of a-synuclein clearance to cerebrospinal fluid [163].

## 5. Proteotoxicity in Alzheimer’s Disease

Alzheimer’s disease (AD) is a neurodegenerative disease characterized by the accumulation of extracellular amyloid-β (Aβ) plaques and intracellular neurofibrillary tangles of hyperphosphorylated tau proteins leading to neuronal loss and cerebral atrophy. AD is the main cause of dementia and accounts for up to 60–80% of all dementia diagnoses [164]. Moreover, the number of new cases with AD increases with age, doubling in prevalence every 5 years after age 65 [165]. Although the vast majority of AD cases are considered sporadic (>90%), they are thought to be influenced by a combination of genetic and environmental factors. Mendelian forms linked to an autosomal dominant pattern of inheritance, are caused by mutations in three genes- amyloid precursor protein (*APP*), presenilin 1 (*PSEN1*), and presenilin 2 (*PSEN2*), representing less than 1% of AD cases [166]. Clinically, AD is defined by progressive cognitive decline enough to affect abilities to work and/or to complete basic daily activities. Behavioral change, impaired mobility, hallucinations and seizures also may emerge with the disease progression. Death is on average 8.5 years from symptoms presentation [167].

### 5.1. Structure of the Amyloid-β Peptide

Amyloid-β (Aβ) peptide is a 39–42 residue protein generated from the sequential processing of the transmembrane amyloid precursor protein (APP) by β- and γ-secretases. First, APP is cleaved by β -secretase producing the membrane-bound fragment C99, which then is subjected to a secondary cleavage by γ-secretase leading to the release of soluble Aβ peptides [168]. The most prevalent forms are peptides with 40 (Aβ1–40) and 42 (Aβ1–42) amino acid residues, the latter being considered as the more toxic species that contributes to the amyloid fibril formation. These species seem to play a key role in amyloid nucleation. A large number of experimental studies have focused on identifying the on-pathway intermediaries responsible for fibril formation. Structural analyses by atomic resolution of Aβ1–42 amyloid fibrils show that the fibril core would be composed of a dimer of Aβ42 molecules. They seem to be arranged resulting in two hydrophobic clusters sited at the end of the chain by a salt bridge [169]. Furthermore, it is likely that a hydrophobic environment favors the formation of prefibrillar aggregates based in intermolecular β-sheet structures, acting as precursors for the amyloid nucleation [170,171]. Apart from these arrangements, the presence of charged residues in the Aβ N-terminal region influences directly the amyloidogenesis process through their interactions and binding with the membrane lipid bilayer. The accumulation of Aβ peptides on membranes would trigger a loss of biophysical properties and subsequently fragmentation [172,173].

### 5.2. Physiological Role of the Amyloid Precursor Protein (APP) and Aβ Peptide

Despite that APP has been largely studied as a precursor of Aβ and a key peptide in the pathogenesis of AD, its physiological functions are not still completely understood. Within the cell, APP is mainly located in the somatodendritic and axonal compartments, and highly abundant in the synaptic terminals. Once it is synthesized in the endoplasmic reticulum, APP matures through the secretory pathway until it reaches the cell surface [174]. Here, it can be cleaved by secretases following two canonical processing pathways, including the amyloidogenic and non-amyloidogenic pathways. The way in which APP is finally processed seems to be linked to its cell localization, thus APP accumulated in the cell surface promotes non-amyloidogenic processing whereas APP stored in intracellular compartments does amyloidogenic processing [175]. Even so, the APP catalytic processing is highly regulated in the cell, and under physiological conditions the non-amyloidogenic pathway is the predominant route. Based on recent investigations, APP is considered as a multimodal protein that can regulate biological processes ranging from transcriptional regulation to synaptic functions. This variety of functions is mediated by cell surface APP or by one its proteolytic fragments [176]. The structural features of APP, which mainly include two heparin-binding domains within the E1 and E2 domains in the extracellular region, strengthens the idea that APP may act as a cell surface receptor. These two domains, with different binding affinities for ligands, would allow the receptor to initiate more rapid and selective intracellular signaling cascade. Moreover, its transmembrane domain could serve as guide signals to carry out the protein transport from endoplasmic reticulum to plasma membrane [177]. As mentioned above, the high expression of APP in synaptic terminals also suggests a role for this protein in synaptic plasticity, learning, and memory. Secreted APP (sAPP) has been proposed to facilitate long-term potentiation and to increase memory in rats and transgenic models of AD [178]. sAPP induces several intracellular signaling cascades that support synaptic plasticity, however, its main receptor target remains unclear. Recently, the gamma-aminobutyric acid type B receptor subunit 1a (GABABR1a) has been identified as synaptic receptor for sAPP. sAPP-GABABR1a binding suppresses synaptic vesicle release and thus modulates synaptic plasticity and neurotransmission [179]. Furthermore, Aβ peptides can exert an opposite effect on synaptic plasticity and memory depending on its concentration. It has been reported that high levels of oligomeric Aβ42 in mouse hippocampal neurons induce synaptic dysfunction leading to memory impairment in AD. However, when picomolar concentrations are used, they promote long-term potentiation (LTP) and memory [180,181].

### 5.3. Pathological Mechanisms of β-Amyloid Peptide Aggregation

Although the pathogenesis of AD is quite complex, there is solid evidence to support that Aβ plays a central role. Aβ42 underlies aggregation when monomer concentration exceeds a critical concentration. This process would act as primary nucleation pathway for the formation of oligomers, and these, in turn, might serve as basis for a slow aggregation into fibrils [182]. Recent studies show a correlation between soluble Aβ oligomer levels and disease severity, highlighting them as the main toxic species [75,183]. Extracellular Aβ oligomers can be formed in the presence of GM1 ganglioside on the cell membrane. These seem to induce neuronal cell death mediated by nerve growth factor (NGF) receptors. Binding of Aβ oligomers to other receptors, such as cellular prion protein and NMDA-type glutamate receptor, causes failure of the neuron signal-transduction pathways related to synaptic function and abnormal calcium homeostasis, respectively. Moreover, Aβ oligomers can induce the loss of insulin receptor from the neuronal surface and impair kinase activity related to long-term potentiation [184]. At the intracellular level, Aβ oligomers inhibit proteasome function resulting in pathological accumulation of Aβ and Tau proteins and subsequently in cell death. The autophagy-lysosome pathway is also impaired. Aβ can accumulate in lysosome and alter its membrane permeability, occasioning the Aβ release to the cytosolic compartment [185,186]. Aβ-induced mitochondrial dysfunction has been extensively associated to AD pathology. This toxic effect would be mediated by a reduction in the electron transport chain enzymatic activities [186,187].

### 5.4. Tau and Phosphorylated Tau

Tau is clearly a determinant protein in the neurodegenerative process of AD [188]. The close association that exists between disease severity and tau load which is higher than those found in Aβ, encourages us to think in a primary role for Tau within the pathological events that occur in AD. Emerging investigations support the idea that Aβ initiates the onset of AD while Tau mediates the subsequent functional deficits at an early stage of the disease. Tau comprises of four domains: N-terminal domain; proline-rich domain, which is located in the central region of the protein; microtubule-binding domain (MBD), which is the region responsible for binding of Tau with microtubules (MTs); and C-terminal domain. The MBD includes four repeats (4R isoform) or three repeats (3R isoform) of approximately a sequence of 32 amino acid residues, as a consequence of alternative splicing in the molecule [189]. These two isoforms are maintained in equilibrium in adult human brains, being essential for a normal function of the protein. One of the widely known roles for Tau is to promote MTs assembly and stability. Interaction of Tau with MTs depends on its phosphorylation/dephosphorylation state by activity of kinases and phosphatases, respectively, binding to MTs when it is dephosphorylated, and separating from them when it is phosphorylated [190]. The interplay between Tau and motor proteins allows the correct axonal transport in the brain, and regulates the development of axonal neurites [191]. Moreover, Tau is also distributed in dendrites at the postsynaptic compartment, suggesting an important role in regulating normal synaptic function [192]. Other recent studies describe a protective role for Tau in the damage of neuronal DNA and RNA by oxidative stress [193], as well as its ability to regulate brain insulin signaling [194]. Despite its low tendency to accumulation, certain post-translational modifications in Tau such as hyperphosphorylation, promote a progressive self-assembly into soluble oligomeric forms and insoluble tangles of filaments [195]. Although the identification of Tau species responsible for neurotoxicity is still under investigation, it is known that pathological aggregation of this protein acts endangering the physiological functions above mentioned and subsequently cell survival [70,196].

### 5.5. Therapeutic Insights

Despite its high prevalence, there is currently no cure or available treatment to significantly modify the progress of the disease. A substantial part of treatments under clinical trial investigation is based on immunotherapy, in which most strategies are directed to Aβ targeting. Whilst it has shown promising results in animal models of AD, human trials are producing disappointing outcomes. There are more than forty immunotherapies (comprising active and passive) registered in the database Alzforum.org (https://www.alzforum.org/therapeutics). However, many of them are not making progress due to unsuccessful clinical efficacy and major safety problems. At present only four have reached Phase 3 of clinical research. Preliminary results after treatment with aducanumab (monoclonal antibody that selectively reacts with Aβ aggregates) have showed a reduction of brain Aβ plaques together with a slowing of clinical decline in patients with prodromal or mild AD [197]. Likewise, administration of gantenerumab (another monoclonal antibody that binds with high affinity to aggregates Aβ species) has resulted in significant Aβ plaques removal at 104 weeks of treatment in patients with prodromal to moderate AD dementia [198]. The ongoing analyses augur to provide valuable information about clinical benefits. Apart from immunotherapy, other anti-amyloid drugs such as β-/γ-secretase inhibitors and γ-secretase modulators are being evaluated. For instance, tarenflurbil has been reported to be a selective Aβ42-lowering agent and also seems to protect from cytotoxicity associated with exposure to Aβ42 in cultured neuronal cells [199,200]. Furthermore, there are therapeutic approaches that are focused on other pathological events, such as synaptic loss, autophagy impairment, mitochondrial dysfunction, and neuroinflammation [201]. However, with the exception of the acetylcholinesterase inhibitors already approved (Donepezil, Rivastigmine, or Galantamine, among others) the majority of treatments are not showing significant clinical benefits or are still in preclinical stages. The complex nature of this disease possibly determines the failure of many therapies directed to one single target. The development of therapies focused on multiple targets might be the base of future strategies.

## 6. Concluding Remarks and Future Directions

Neurodegenerative diseases are a great burden for our health system. This review has examined the advances that have been made in understanding how molecular mechanisms get disrupted by proteotoxicity causing cell death in these diseases. We have discussed how disease arises as a result of neural cells not being capable of maintaining the stability of its proteome through adequate folding and degradation of aberrant proteins. Despite efforts, our knowledge of disease aetiology is still limited and so far it is unknown whether aggregation is a cause or a protective consequence for disease. This review supports the notion that although shared features driving proteotoxicity are implicated in these four neurodegenerative diseases including protein misfolding and aggregation, protein propagation, and proteostasis impairment, the current literature suggests that the proteins and aggregates implicated in each of these diseases as well as the genetic basis driving their pathology are not shared but disease specific. Additional evidence to unravel the coexistence of proteins and their interactions among these neurodegenerative diseases is required. Along the way we have learnt that therapeutic strategies designed to prevent or stop disease progression will not be possible without critically re-establishing perturbations in protein quality control. Increasing proteasome activity, enhancing cellular PQC mechanisms by upregulation of chaperones and clearance cascade pathways, as well as controlling the capability of misfolded proteins to propagate to nearby cells, are key to avoid progressive neuronal degeneration. As highlighted in this review, successful treatment opportunities will not be sufficient with single-target approaches aimed to specifically tag toxic protein species. The new era for the treatment of proteinopathies holds promise while we continue unraveling the pathological processes underlying these complex diseases.

## Figures and Tables

**Table 1:** Proteotoxicity hallmarks across neurodegenerative diseases.

Neurodegenerative Disease	Protein Aggregates	IDR Protein Structure	Species Location	Toxicity	References
Huntington’s disease	Huntingtin	PolyQ	Intracellular (cytosolic and nuclear)	Plasma-membrane integrity disruption Transcriptional dysregulation Reduced levels of neurotrophic factors as BDNF Impairment of protein degradation systems Mitochondrial dysfunction Reactive gliosis Neuroinflammation Cell death	[4] [5] [6] [7,8] [9,10] [11] [12] [13]
Amyotrophic Lateral Sclerosis	TPD-43	C-Terminal Domain	Cytoplasmic aggregate	Affected mRNA splicing and RNA metabolism proteins Global protein synthesis inhibition Mitochondrial impairment Defective autophagy lysosomal pathway Endocytosis impairment Dysregulated metal ions (as zinc and manganese) Alteration in chromatin dynamics	[14,15] [16] [17,18] [19,20,21] [22,23] [24,25] [26]
	FUS	N-Terminal domain		Affected mRNA metabolism and DNA reparation Defects in Protein Quality Control (PQC) system	[27] [28]
	SOD-1	22–30,55–95 region 121–143 region		Excitotoxity linked to glutamate transporter EAAT2 Excessive calcium influx Mitochondrial dysfunction Compromised axonal transport ROS cytotoxicity RNA species damaged	[29,30] [29,30] [31,32,33] [34,35,36] [37] [38]
	Ataxin-2	PolyQ tract		Stress response dysfunction Affected RNA metabolism	[39] [40]
	TBK-1	TBK-1		Autophagy dysfunction	[41]
Parkinson’s disease	α-Synuclein	C-terminal domain	Intracellular LBs formation, extracellular and membrane	Plasma-membrane integrity disruption Synapse alteration Perturbation in calcium homeostasis Cytoskeleton dynamics altered Protein degradation system dysfunction Lysosomal impact Mitochondrial dysfunction and ROS induction Endoplasmic reticulum stress Golgi transmission affected Modified histone acetylation procedures Apoptosis	[42,43,44,45] [46,47] [48,49] [50,51,52] [53,54,55] [55,56,57] [58,59] [59,60] [61,62] [63,64,65] [66,67]
Alzheimer’s disease	Amyloid-β	Amyloid-β	Extracellular plaques	Plasma-membrane alteration Perturbed synaptic function and structure Glial cells perturbation via mGluR5 receptor Altered calcium homeostasis LTP inhibition in the CA1 region of the hippocampus Oxidative stress disfunction	[68,69] [70,71,72] [73] [74] [75,76] [77,78]
	Tau	N-terminal domain	Intracellular neurofibrillary tangles	Telomerase dysfunction Mitochondrial damage and ROS Lipid peroxidation Activated microglia leading to neuronal phagocytosis Apoptosis	[79,80] [81,82] [83] [84,85] [86]

## Abbreviations

AD	Alzheimer’s disease
PD	Parkinson’s disease
ALS	Amyotrophic lateral sclerosis
FTD	Frontotemporal dementia
HD	Huntington’s disease
PQC	Protein quality control
UPS	Ubiquitin-proteasome system
polyQ	Polyglutamine
BDNF	Brain-derived neurotrophic factor
AAV	Adeno-associate virus
ASOs.	Antisense oligonucleotides
SOD1	Superoxide Dismutase 1
FUS	Fused in Sarcoma
TBK-1	TANK-binding kinase 1
hnRNP	Ribonucleoprotein family
TPD-43	TAR DNA-binding protein 43
NTD	N-Terminal Domain
RRMs	RNA Recognition Motifs
NES	Nuclear Export Signal
NLS	Nuclear Localization Signal
CTD	C-Terminal Domain
CTF	C-terminal fragments
RBP	RNA binding protein
ROS	Reactive oxygen species
Q	Glutamines
NLS	C-terminal nuclear localization signal
CTF	C-terminal fragments
RBP	RNA binding protein
KD	Kinase domain
ULD	Ubiquitin-like domain
CCD1 and CCD2	Two coiled-coil domains
AIM4	Acridine-imidazole derivative
LB	Lewy bodies
LN	Lewy neurites
ER	Endoplasmic reticulum
ALP	Autophagy lysosomal pathway
AB	Amyloid-beta
APP	Amyloid precursor protein
PSEN1	Presenilin 1
PSEN2	Presenilin 2
APP	Amyloid precursor protein
sAPP	Secreted APP
GABABR1a	Gamma-aminobutyric acid type B receptor subunit 1a
LTP	Promote long-term potentiation
NGF	Nerve growth factor
MDR	Microtubule-binding domain
